# Validity and reliability of the arabic version of the self-report single-item self-esteem scale (A-SISE)

**DOI:** 10.1186/s12888-023-04865-y

**Published:** 2023-05-22

**Authors:** Feten Fekih-Romdhane, Zeinab Bitar, Radosław Rogoza, Abir Sarray El Dine, Diana Malaeb, Tabassum Rashid, Sahar Obeid, Souheil Hallit

**Affiliations:** 1 The Tunisian Center of Early Intervention in Psychosis, Department of psychiatry “Ibn Omrane”, Razi Hospital, Manouba, 2010 Tunisia; 2 Faculty of Medicine of Tunis, Tunis El Manar University, Tunis, Tunisia; 3 University of Economics and Human Sciences in Warsaw, Warsaw, Poland; 4 Department of Psychology, University of Lleida, Lleida, Spain; 5 Department of Biomedical Sciences, School of Arts and Sciences, Lebanese International University, Beirut, Lebanon; 6 School of Pharmacy, Lebanese International University, Beirut, Lebanon; 7 College of Pharmacy, Gulf Medical University, Ajman, United Arab Emirates; 8 Psychology Department, College of Humanities, Effat University, Jeddah, 21478 Saudi Arabia; 9 Social and Education Sciences Department, School of Arts and Sciences, Lebanese American University, Jbeil, Lebanon; 10 School of Medicine and Medical Sciences, Holy Spirit University of Kaslik, P.O. Box 446, Jounieh, Lebanon; 11 Applied Science Research Center, Applied Science Private University, Amman, 11931 Jordan; 12 Faculty of Medicine, Paris-Saclay University, Le Kremlin-Bicêtre, France; 13 Research Department, Psychiatric Hospital of the Cross, Jal Eddib, Lebanon

**Keywords:** Single-item self-esteem scale, SISE, Arabic, Psychometric properties, Validation

## Abstract

**Background:**

Meta-analytic findings documented a substantial impact of self-esteem on a broad range of psychological and behavioral indicators, thus highlighting its high clinical relevance. Proving a simple and cost-effective measure of global self-esteem to the Arabic-speaking community, who mostly live in low- and middle-income countries, and where research may be challenging, would be highly valuable. In this context, we sought to investigate the psychometric characteristics of an Arabic translation of the Single-Item Self-Esteem Scale (A-SISE) in terms of factor structure, reliability, and construct validity.

**Methods:**

A total of 451 participants were enrolled between October and December 2022. An anonymous self-administered Google Forms link was shared on WhatsApp. To examine the factor structure of the A-SISE, we used the FACTOR software. We conducted an exploratory factor analysis (EFA), using a principal component analysis on the Rosenberg Self-Esteem Scale (RSES) items first, then after adding the A-SISE.

**Results:**

The results of the EFA of the RSES revealed two factors (F1 = negatively-worded items; F2 = positively-worded items), which explained 60.63% of the common variance. When adding the A-SISE, the two-factor solution obtained explained 58.74% of the variance, with the A-SISE loading on the second factor. Both RSES and A-SISE correlated significantly and positively with each other, as well as with extroversion, agreeableness, conscientiousness, open mindedness and satisfaction with life. Moreover, they correlated significantly and negatively with negative emotionality and depression.

**Conclusion:**

These results suggest that the A-SISE is a simple-to-use, cost-effective, valid and reliable measure of self-esteem. We thus recommend its use in future research among Arabic-speaking people in Arab clinical and research settings, particularly when researchers are limited by time or resources constraints.

## Background

Self-esteem represents an evaluative component of the self-concept [1], in which individuals evaluate their self-image based on feedback and information they get through social interaction during their various social roles [2]. Self-esteem is significant on both personal and social levels; it is seen as a personal psychological characteristic [3] that involves an awareness of one’s value system and an emotional assessment of one’s own worth [4]. Global self-esteem plays a protective role in the person’s system [5]; and constitutes an essential element in personal well-being [6]. Meta-analytic findings documented a substantial impact of self-esteem on a broad range of psychological and behavioral indicators, including depression, anxiety [7], social anxiety [8], social relationships [9], body dysmorphic disorder symptom severity [10], feelings of shame [11], problematic smartphone use [12], eating disorders [13], sexual functioning [14], academic achievements [15], suicidal behavior [16], peer victimization [17], cyberbullying [18], bullying perpetration [19], aggression [20], as well as crime and delinquency [21]. In addition, prospective reports have shown that low levels of self-esteem experienced in adolescence have long lasting effects on both physical and mental health in adulthood and later life [9, 22–24]. Self-esteem has also proven to be a major and impactful predictor of quality of life [25] and satisfaction with life [26]. Therefore, investigating and studying self-esteem is of high relevance and importance in the psychological and psychiatric fields. To this end, it is important to have a self-esteem assessment tool that adapts to each environment, community and has appropriate psychometric characteristics.

### Measures of self-esteem: the Rosenberg self-esteem scale

The most common instrument used to assess self-esteem is the Rosenberg self-esteem scale (RSES). It was developed in 1965 by Morris Rosenberg [27] who considered self-esteem as person’s thoughts and feelings about self-worth [27]. The RSES is measured by ten items answered on a four-point scale with responses ranging from strongly disagree (1) to strongly agree (4) [27]. Half of the items are positively formulated (e.g. “I feel that I have a number of good qualities”), while the other half is negatively formulated (e.g. “I wish I could have more respect for myself”) [27]. The RSES is a reliable and valid scale of global self-worth, that has been adapted across 53 nations and translated to almost 28 languages [28] such as Persian [29], French [30], Chinese [31], Italian [32], Estonian [33], Portuguese [34], Spanish [5], German [35], Greek [36], and Arabic [37]. However, over the last decades, the psychometric properties of the RSES have been questioned internationally. Some of these translational validation studies revealed inconsistencies in replicating the original unidimensional factor structure of the RSES [28]. Few studies presented the scale as unidimensional [27, 38], while others suggested a two-dimensional structure [39]. The first factor, grouping the positively formulated items, refers to the positive self-esteem, or positive self-worth and the second factor, grouping the negatively formulated items, refers to the negative self-esteem, or self-depreciation [33]. Nevertheless, no consensual interpretation of the two-dimensions is yet available; with studies describing them either as methodical artefacts of item-wording (e.g., [40]) or as related but separated aspects of the self (e.g., [41]). This might significantly affect the understanding and interpretation of the self-esteem construct and its related scores. Another issue related to the RSES has been raised by cross-national research; with negatively worded items having been demonstrated to be interpreted differently depending on the cultural and country context [28].

### Measures of self-esteem: the single-item self-esteem scale

More recently, Robins et al. [42] designed an ultra-brief measure to assess global self-esteem, i.e. the Single-Item Self-Esteem Scale (SISE). Due to their briefness, self-report single-item scales are practical and easy to administer for both clinicians and researchers, particularly in large exploratory or field studies and those using multi-point assessments where participants’ time and burden and survey costs need to be accounted for. Apart from their convenient usefulness, single-item measures have consistently been shown to be valid and reliable; thus leading to the growing recommendation of their usage and to their gradual inclusion in guidelines [43]. In addition to these potential advantages, single-item scales are psychometrically sound, given that analysis of data from Likert-type format of responses at the item level is statistically solid [44]. Examples of constructs that have previously been reliably and validly assessed using single-items scales include narcissism [45], risk-taking [46], Fear of Missing Out [47], job satisfaction [48], and social identification [49]. For self-esteem, however, the single-item measure received scant research interest. The original validation study performed in the English language among US undergraduate students has evidenced the validity and reliability of the SISE, and showed that the SISE and the RSES exhibited similar correlation patterns with outcome measures including depression, life satisfaction, and maladaptive personality traits [42]. Since then, the SISE has then been validated in two other languages, i.e. German [50] and Brazilian [51]; with both versions supporting its adequate psychometric properties. However, no Arab version of the SISE exits to date, to the best of our knowledge.

### The present study

Self-esteem appears to be a culturally-dependent concept, having its roots in Western societies [52]. It stems from the individualistic culture of the West which places priority on the autonomy and power of individuals [53], and therefore a stronger emphasis on cultivating positive self-esteem. In countries with individuated cultural values (e.g., the United States), all individuals are taught since the childhood to stand up for themselves and view themselves as special [54]. They all possess a “self”, and self-esteem is considered a “basic human need” [55]. Cross-cultural research showed that the self is viewed differently between cultures, and self-esteem vary widely between individualistic and collectivistic societies (e.g., [56]). Arab societies and cultures are collectivist in nature [57], where the self is perceived as interdependent and merged with the members of the in-group [58]. Within-cultures differences in self-esteem have also been described. For instance, a cross-national comparison by Abdel-Khalek et al. [59] found that Kuwaiti and Omani college students displayed greater levels of self-esteem compared with their counterparts from Egyptian and Lebanese. These variations were partly explained by differences in per-capita income levels and employment possibilities between countries that are suggested to affect self-esteem [59]. It is of note, however, that a dearth of literature on self-esteem emerged from the Arab world to date (e.g., [60]). To help foster national and cross-national research on this topic in Arab countries, we sought to investigate the psychometric characteristics of an Arabic translation of the SISE (A-SISE) in terms of factor structure, reliability, and construct validity. Patterns of associations between A-SISE and depression symptoms, satisfaction with life, and personality traits have also been examined. Proving this simple and cost-effective measure of global self-esteem to the Arabic-speaking community, who mostly live in low- and middle-income countries, and where research may be challenging, would be highly valuable.

## Methods

### Participants

Four hundred fifty one Lebanese citizens and residents (women n = 292; 64.7%) completed the survey, who had a mean age of 23.58 years (SD = 9.44). The majority of participants were single (86.3%), while 85.8% had a university level of education (Table 1).

**Table 1:** Sociodemographic and other characteristics of the participants (N = 451)

Variable	N (%)
**Sex**	
Male	159 (35.3%)
Female	292 (64.7%)
**Marital status**	
Single	389 (86.3%)
Married	62 (13.7%)
**Education level**	
Secondary or less	64 (14.2%)
University	387 (85.8%)
	**Mean ± SD**
**Age (in years)**	23.58 ± 7.98

### Minimum sample size

Following the recommendations of Comrey and Lee [61], a minimum sample of 10 participants per scale’s item are needed to conduct an exploratory factor analysis. Since the A-SISE is composed of one question, a minimal sample of 10 participants was needed.

### Measures

The Arabic questionnaire assessed the sociodemographic characteristics of the included participants (age, sex, marital status and education), as well as the following scales:

**Self-Esteem.** Two measures of self-esteem were used; (1) the A-SISE (English: “I have high self-esteem”) rated on a 5-point Likert scale (1 = not at all true of me, 2 = rather not true of me, 3 = some part true of me, 4 = rather true of me, 5 = very true of me), and (2) the Arabic version of the Rosenberg self-esteem scale [27, 37]: It is a 10-item scale that reflects self-worth by focusing on both positive and negative feelings people have about themselves. Items are scored on a four-point Likert scale (1 = strongly disagree to 4 = strongly agree). Higher scores reflect a better self-esteem. The forward and backward translation method was applied to the SISE following international guidelines [62]. The English version was translated to Arabic by a Lebanese translator who was completely unrelated to the study. Afterwards, a Lebanese psychologist with a full working proficiency in English, translated the Arabic version back to English. The initial and translated English versions were compared to detect and later eliminate any inconsistencies by a committee composed of the research team and the two translators [63, 64]. A pilot study was conducted on 20 persons before the start of the official data collection to make sure the question was well understood; no changes were done consequently.

**Satisfaction with life Scale**. This measure has been validated in Lebanon [65]. It is composed of 5 items, rated on a 7-point Likert scale (1 = strongly disagree to 7 = strongly agree). Higher scores reflect a higher satisfaction with life (ω = 0.88).

**Big-Five Inventory (BFI-2) extra short form** [66] was used to assess personality traits. This latest version comprises 15 questions, rated on a scale from 1 = strongly disagree to 5 = strongly agree, which yields 5 personality traits: extroversion (ω = 0.36), agreeableness (ω = 0.43), conscientiousness (ω = 0.46), negative emotionality (ω = 0.60) and open mindedness (ω = 0.29). The Arabic translation of the BFI-2 has directly been obtained from Dr Soto (the developer of the scale), and has previously been used in validation research in the Lebanese population [67].

The Arabic version [68] of the **Hamilton Depression scale 7 items** was used to assess depression. It is a shorter form of the 17-item HAM-D scale [69], already validated in Arabic [70] (ω = 0.72).

### Procedures

Between October and December 2022, all information was gathered by way of a Google Form link. The research team employed the “snowball” approach, in which they made contact with people they know and requested them to share the link with their friends and relatives. A projected completion date was included in the project’s social media advertising. Being an adult resident and citizen of Lebanon was a requirement for participation. To make sure no one completed the poll more than once, Internet protocol (IP) addresses were checked. The above-mentioned items were administered in a pre-randomized order to account for order effects after participants had provided digital informed permission. Participants freely completed the survey, which was anonymous, and without remuneration.

### Analytic Strategy

#### Data treatment

There were no missing responses in the dataset. To examine the factor structure of the A-SISE, we used an exploratory factor analysis, using a principal component analysis using the FACTOR software [71]. We verified all requirements related to item-communality [72], average item correlations, and item-total correlations [73]. The Kaiser-Meyer-Olkin (KMO) measure of sampling adequacy (which should ideally be ≥ 0.80) and Bartlett’s test of sphericity (which should be significant) ensured the adequacy of our sample [74]. The procedure followed for determining the number of dimensions was the Parallel Analysis (PA) [75], using the Pearson correlation matrix. Weighted Root Mean Square Residual (WRMR) were also calculated to assess the model fit (values < 1 have been recommended to represent good fit [76]. Item retention was based on the recommendation that items with “fair” loadings and above (i.e., ≥ 0.33) and with low inter-item correlations (suggestive of low item redundancy) as indicated by the anti-image correlation matrix should be retained [77].

#### Further analyses

Composite reliability in both subsamples was assessed using McDonald’s (1970) ω, with values greater than 0.70 reflecting adequate composite reliability [78]. McDonald’s ω was selected as a measure of composite reliability because of known problems with the use of Cronbach’s α (e.g., [79]). The total RSES and the A-SISE scores followed a normal distribution, with skewness and kurtosis values varying between − 1 and + 1 [80]. To assess convergent and criterion-related validity, we examined bivariate correlations between total RSES and the A-SISE scores and those on the additional measures included in the survey (personality traits, depression and satisfaction with life) using the Pearson test. Student t test was used to compare two means. Based on Cohen (1992) [81], values ≤ 0.10 were considered weak, ~ 0.30 were considered moderate, and ~ 0.50 were considered strong correlations.

## Results

### Descriptive statistics

Table 2 presents the descriptive statistics of the used scales, which were all considered as normally distributed. The A-SISE had a mean of 3.72 (SD = 0.96, range: 1–5), a median of 4.00, a mode of 4, and the following score distribution: 1 = 3.3%, 2 = 4.9%, 3 = 29.7%, 4 = 41.0%, 5 = 21.1%.

**Table 2:** Descriptive statistics of all scores

	Mean	SD	Min	Max	Skewness	Kurtosis
A-SISE	3.72	0.96	1	5	− 0.64	0.42
RSES	30.11	4.84	15	40	− 0.12	− 0.16
SWL	19.45	6.80	5	35	0.07	− 0.89
Depression	5.73	4.24	0	21	0.64	0.19
Extroversion	9.13	1.81	4	14	− 0.02	0.22
Agreeableness	11.06	1.90	4	15	− 0.33	0.08
Conscientiousness	10.48	2.12	5	15	− 0.04	− 0.53
Negative emotionality	9.57	2.48	3	15	0.01	− 0.18
Open mindedness	9.92	1.66	4	15	− 0.01	0.11

### Exploratory factor analysis

#### Factor analysis on the total sample

Bartlett’s test of sphericity, χ^2^(45) = 1752, *p* < .001, and KMO (0.862) indicated that the RSES items had adequate common variance for factor analysis. The results of the EFA revealed two factors, which explained 60.63% of the common variance. The WRMR value was also adequate (= 0.073; 95% CI 0.066-0.078), indicating good fit of the model.

When adding the A-SISE, Bartlett’s test of sphericity, χ^2^(55) = 1977.3, *p* < .001, and KMO (0.874) remained adequate. The two-factor solution obtained explained 58.74% of the variance (WRMR = 0.07; 95% CI 0.063-0.075).

#### Factor analysis on with men

Similar results were seen in men; Bartlett’s test of sphericity, χ^2^(45) = 772.2, *p* < .001, and KMO (0.824) again indicated that the RSES scales’ items had adequate common variance for factor analysis among men. A two-factor solution was obtained explaining 64.76% of the variance (WRMR = 0.069; 95% CI 0.055-0.078).

When adding the A-SISE, Bartlett’s test of sphericity, χ^2^(55) = 890.7, *p* < .001, and KMO (0.843) remained adequate. The two-factor solution obtained explained 63.58% of the variance (WRMR = 0.067; 95% CI 0.053-0.076).

#### Factor analysis with women

For women, Bartlett’s test of sphericity, χ^2^(45) = 1087, *p* < .001, and KMO (0.862) again indicated that the RSES items had adequate common variance for factor analysis. The results of the EFA revealed two factors, which explained 60.22% of the common variance (WRMR = 0.071; 95% CI 0.060-0.078), indicating good fit of the model.

When adding the A-SISE, Bartlett’s test of sphericity, χ^2^(55) = 1205.8, *p* < .001, and KMO (0.874) remained adequate. The two-factor solution obtained explained 57.92% of the variance (WRMR = 0.068; 95% CI 0.058-0.074).

#### Factor structure congruence and composite reliability

The factor loadings reported in Table 3 for the total sample, women and men separately suggest strong similarity across factor structures. McDonald’s ω were very good for each of the two factors of the RSES and for the A-SISE in the total sample, men and women respectively.

**Table 3:** Rotated factor loads obtained from the Exploratory Factor Analysis (EFA)

EFA 1: conducted on the total sample.				
	Model 1: EFA of RSES items alone		Model 2: EFA of RSES items + A-SISE	
	Factor 1	Factor 2	Factor 1	Factor 2
RSES 1. On the whole, I am satisfied with myself.	− 0.04	**0.77**	− 0.02	**0.79**
RSES 2. At times I think I am no good at all.	**0.77**	− 0.12	**0.77**	− 0.11
RSES 3. I feel that I have a number of good qualities	0.12	**0.83**	0.15	**0.83**
RSES 4. I am able to do things as well as most other people.	− 0.02	**0.79**	0.01	**0.79**
RSES 5. I feel I do not have much to be proud of.	**0.70**	− 0.04	**0.70**	− 0.03
RSES 6. I certainly feel useless at times.	**0.81**	− 0.06	**0.81**	− 0.04
RSES 7. I feel that I’m a person of worth, at least on an equal plane with others.	0.04	**0.78**	0.07	**0.78**
RSES 8. I wish I could have more respect for myself.	**0.74**	0.03	**0.75**	0.26
RSES 9. All in all, I am inclined to feel that I am a failure.	**0.76**	− 0.04	**0.76**	− 0.05
RSES 10. I take a positive attitude toward myself.	− 0.08	**0.78**	− 0.06	**0.79**
A-SISE. I have high self-esteem.	-	-	− 0.28	**0.51**
McDonald’s ω	0.81	0.84	0.81	0.83
**EFA 2: conducted on men only.**				
RSES 1. On the whole, I am satisfied with myself.	− 0.06	**0.84**	− 0.02	**0.86**
RSES 2. At times I think I am no good at all.	**0.82**	− 0.12	**0.81**	− 0.11
RSES 3. I feel that I have a number of good qualities	0.10	**0.84**	0.13	**0.83**
RSES 4. I am able to do things as well as most other people.	0.06	**0.85**	0.10	**0.85**
RSES 5. I feel I do not have much to be proud of.	**0.78**	0.06	**0.78**	0.07
RSES 6. I certainly feel useless at times.	**0.81**	− 0.14	**0.80**	− 0.13
RSES 7. I feel that I’m a person of worth, at least on an equal plane with others.	− 0.07	**0.75**	− 0.04	**0.76**
RSES 8. I wish I could have more respect for myself.	**0.63**	0.38	**0.65**	0.37
RSES 9. All in all, I am inclined to feel that I am a failure.	**0.73**	− 0.08	**0.73**	− 0.11
RSES 10. I take a positive attitude toward myself.	− 0.04	**0.85**	− 0.002	**0.85**
A-SISE. I have high self-esteem.	-	-	− 0.20	**0.68**
McDonald’s ω	0.80	0.87	0.80	0.87
**EFA 3: conducted on women only.**				
RSES 1. On the whole, I am satisfied with myself.	− 0.06	**0.72**	− 0.05	**0.74**
RSES 2. At times I think I am no good at all.	**0.78**	− 0.09	**0.78**	− 0.08
RSES 3. I feel that I have a number of good qualities	0.14	**0.84**	0.17	**0.84**
RSES 4. I am able to do things as well as most other people.	− 0.08	**0.75**	− 0.06	**0.75**
RSES 5. I feel I do not have much to be proud of.	**0.66**	− 0.10	**0.66**	− 0.08
RSES 6. I certainly feel useless at times.	**0.84**	0.01	**0.83**	0.02
RSES 7. I feel that I’m a person of worth, at least on an equal plane with others.	0.10	**0.81**	0.12	**0.81**
RSES 8. I wish I could have more respect for myself.	**0.75**	0.18	**0.76**	0.18
RSES 9. All in all, I am inclined to feel that I am a failure.	**0.78**	− 0.002	**0.79**	0.002
RSES 10. I take a positive attitude toward myself.	− 0.10	**0.77**	− 0.08	**0.77**
A-SISE. I have high self-esteem.	-	-	− 0.31	**0.46**
McDonald’s ω	0.81	0.82	0.81	0.80

Factor 1 = Negatively worded items; Factor 2 = Positively worded items, RSES = Rosenberg Self-Esteem Scale; A-SISE = Arabic Single Item Self-Esteem. Numbers in bold indicate the highest loading of the item on its respective factor.

### Construct validity

Both RSES and A-SISE correlated significantly and positively with each other, as well as with extroversion, agreeableness, conscientiousness, open mindedness and satisfaction with life. Moreover, they correlated significantly and negatively with negative emotionality and depression (Table 4). Finally, a higher mean RSES score, but not A-SISE score, was found in participants with a university level of education compared to secondary or less (Table 5).

**Table 4:** Correlation of the Rosenberg Self-Esteem Scale (RSES) and the Arabic version of the Single Item Self-Esteem Scale (A-SISE) with other continuous variables

	1	2	3	4	5	6	7	8	9	10
1. RSES	1									
2. A-SISE	0.58***	1								
3. Extroversion	0.21***	0.14**	1							
4. Agreeableness	0.24***	0.18***	− 0.04	1						
5. Conscientiousness	0.52***	0.28***	0.18***	0.29***	1					
6. Negative emotionality	− 0.43***	− 0.26***	− 0.17***	− 0.09	− 0.39***	1				
7. Open mindedness	0.28***	0.24***	0.25***	0.18***	0.31***	− 0.17***	1			
8. Depression	− 0.39***	− 0.27***	− 0.13**	− 0.05	− 0.24***	0.55***	− 0.11*	1		
9. Satisfaction with life	0.57***	0.38***	0.22***	0.26***	0.38***	− 0.42***	0.26***	− 0.42***	1	
10. Age	0.03	− 0.03	− 0.05	− 0.15**	0.05	− 0.02	− 0.14**	− 0.05	− 0.06	1

*p < .05; **p < .01; ***p < .001

**Table 5:** Bivariate analysis of the Rosenberg Self-Esteem Scale (RSES) and the Arabic version of the Single Item Self-Esteem Scale (A-SISE) with categorical variables

	RSES			A-SISE		
	Mean ± SD	*p*	Effect size (Cohen’s d)	Mean ± SD	*p*	Effect size (Cohen’s d)
**Sex**		0.212	0.123		0.990	0
Male	30.50 ± 4.93			3.72 ± 1.01		
Female	29.90 ± 4.79			3.72 ± 0.93		
**Marital status**		0.670	0.056		0.443	0.100
Single	30.07 ± 4.82			3.73 ± 0.95		
Married	30.35 ± 5.02			3.63 ± 1.04		
**Education level**		**0.003**	0.390		0.215	0.177
Secondary or less	28.44 ± 5.26			3.55 ± 1.21		
University	30.39 ± 4.72			3.74 ± 0.91		

Numbers in bold indicate significant p values.

## Discussion

We sought through the present study to translate and validate the Arabic version of the single-item measure of global self-esteem, i.e. the A-SISE. EFA confirmed good congruence of factor structure across gender. The A-SISE displayed acceptable composite reliability coefficients. Both RSES and A-SISE revealed comparable associations with investigated variables (life satisfaction, personality traits and depression), which provided sufficient level of construct-validity.

We found a mean A-SISE score of 3.72 ± 0.96. These results are comparable to mean SISE scores found in the US (3.50 ± 1.10, [42]) and German (3.25 ± 1.15, [50]) samples; and slightly lower than those found in the Brazilian student sample (4.36 ± 1.45, [51]). EFA yielded a two-factor structure of the Arabic RSES, and showed that A-SISE loaded on the same factor (Factor 2) as the five positively-worded items of the Arabic RSES. Additionally, we found positive correlations between the RSES and A-SISE scores, suggesting that the single-item scale is informative and relevant to assess the self-esteem construct. We also found that both RSES and A-SISE positively correlated with extroversion, agreeableness, conscientiousness, open mindedness, satisfaction with life, and inversely correlated with negative emotionality and depression severity, thus aligning with findings from earlier studies that investigated self-esteem. In the parent validation, higher SISE scores correlated with greater extraversion, conscientiousness, and less neuroticism [42]. Comparable patterns of associations between SISE scores and personality traits have been observed in the German sample, where self-esteem was positively related to extraversion and conscientiousness, and negatively related to neuroticism [50]. The inverse association between self-esteem (as assessed using the SISE) and depression has also been demonstrated in the original [42] and German validation studies [50]. Finally, the positive link between SISE scores and life satisfaction has been noted in the originally developed version [42]. These findings support the convergence between the Arabic RSES and the A-SISE, and the construct validity of the A-SISE.

Finally, the gender comparison of self-esteem scores revealed no significant differences between men and women, both when using the RSES or the A-SISE. In the original and German validations of the SISE [50], as well as other previous research using different measures (e.g., [82, 83]), male participants generally exhibit significantly higher self-esteem scores than females. However, gender differences in self-esteem have been shown to vary across cultures, and to be more pronounced in Western industrialized high-income countries [84]; which might explain our findings. All these results suggest that the A-SISE is a simple-to-use, cost-effective, valid and reliable measure of self-esteem. We thus recommend its use in future research, particularly when researchers are limited by time or resources constraints.

### Study limitations

When discussing the limitations of the present study, we should start by emphasizing that single-item scales may have their shortages (e.g., reduced psychometric performance [43, 85]) in some contexts or situations compared to multi-item measures. Therefore, it is important to adequately choose the appropriate research settings where to use the A-SISE. In addition, single-item measures have been criticized for having lower/uncertain reliability, as measurement error estimation is expected to not follow the prescribed model which relies on inter-correlations to account for reliability (i.e., the internal consistency approach) [86]. It is thus suggested that, only one item may not allow the measure to be subjected to internal consistency procedures [86]. This can be particularly of concern in our study, as the personality measure used (i.e., the Arabic BFI-2) exhibited low reliability. To address this limitation, alternative methods (e.g., test-retest reliability) have been recommended [86], and need to be considered in future studies. Future validation studies should also consider using measures with adequate reliability when examining construct validity of the A-SISE. Furthermore, it should be noted that all SISE correlations are lower than the correlations found with the Rosenberg scale; this indicates limitations regarding the convergent validity of the single-item measure. Other limitations have also to be discussed. This is a cross-sectional study, which means causation cannot be inferred. A recall bias might be present and it may have led to an overestimation of the answers given to some questions Symptoms were self-reported (not evaluated by a healthcare professional) and thus are subjective. Additionally, results of this study cannot be generalized to the whole population because the sample included a majority of people with a university level of education, who were recruited by using the snowball technique.

## Conclusion

The goal of the current study was to provide evidence of reliability and validity of the A-SISE, which was accomplished through the examination of its congruence of factor structure across gender, composite reliability and construct-validity. Consequently, the single-item scale is deemed a suitable measure to assess self-esteem, and is recommended for use among Arabic-speaking people in Arab clinical and research settings when appropriate.

## Data Availability

All data generated or analyzed during this study are not publicly available to maintain the privacy of the individuals’ identities. The dataset supporting the conclusions is available upon request to the corresponding author.
